# The Complementary Role of Optical Coherence Tomography and Fluorescein Angiography in Diagnosing and Monitoring Retinal Vascular Status in Susac Syndrome: Two Case Reports

**DOI:** 10.3390/reports9020168

**Published:** 2026-05-27

**Authors:** Zuzanna Wilk, Olga Kaczmarek, Sławomir Liberski, Danuta Nikratowicz, Szczepan Cofta, Goran Petrovski, Jarosław Kocięcki

**Affiliations:** 1 Department of Ophthalmology, Poznan University of Medical Sciences, A. Szamarzewskiego 84, 61-848 Poznan, Poland; z.wilk012@gmail.com (Z.W.); olgakaczmarek2000@wp.pl (O.K.); danuta.nikratowicz@usk.poznan.pl (D.N.); j.kociecki@gmail.com (J.K.); 2 Department of Respiratory Medicine, Allergology and Pulmonary Oncology, Poznan University of Medical Sciences, A. Szamarzewskiego 84, 61-848 Poznan, Poland; s.cofta@gmail.com; 3 Center for Eye Research and Innovative Diagnostics, Department of Ophthalmology, Institute for Clinical Medicine, University of Oslo, Kirkeveien 166, 0450 Oslo, Norway; goran.petrovski@medisin.uio.no; 4 Department of Ophthalmology, Oslo University Hospital, Kirkeveien 166, 0450 Oslo, Norway

**Keywords:** Susac syndrome, SICRET syndrome, microangiopathy, retinal complications

## Abstract

**Background and Clinical Significance**: Susac syndrome is a rare autoimmune-mediated microangiopathy characterized by the triad of encephalopathy, branch retinal artery occlusion (BRAO), and sensorineural hearing loss. Due to its variable onset and protean manifestations, the syndrome is frequently misdiagnosed, potentially leading to delayed treatment and irreversible organ damage. Ocular involvement is common and often provides the first diagnostic clue. Multimodal imaging, particularly fluorescein angiography (FA) and optical coherence tomography (OCT) as well as optical coherence tomography angiography (OCT-A), enables the detection of both acute and chronic ischemic retinal changes. Their complementary application yields critical insights into disease activity, supports monitoring of relapses, and guides therapeutic strategies. **Case Presentation**: We describe two patients with Susac syndrome presenting with distinct ocular and neurological features. A 43-year-old male developed recurrent BRAOs in both eyes, documented by FA, OCT, and OCT-A, with preserved best-corrected visual acuity (BCVA) of 0.00 logMAR in both eyes (OU). OCT demonstrated progressive thinning of the retinal nerve fiber layer (RNFL) and inner retinal layers, consistent with sequelae of microinfarctions, while FA revealed focal arteriolar wall hyperfluorescence. Immunosuppressive therapy with corticosteroids and mycophenolate mofetil stabilized his condition. A 31-year-old female with a history of migraine and encephalopathy showed thinning of the RNFL and ganglion cell layer (GCL) with macular atrophy on OCT. FA demonstrated peripheral arteriolar wall hyperfluorescence and microaneurysms. Despite these structural alterations, visual acuity remained unaffected. Serial imaging initially demonstrated mild progression on OCT and OCT-A, followed by disease stabilization under systemic immunosuppressive therapy. **Conclusions**: These cases highlight the pivotal role of multimodal imaging in the early recognition and long-term monitoring of Susac syndrome. OCT provides a detailed assessment of retinal microinfarctions and chronic atrophy, while FA remains indispensable for detecting vascular leakage and disease activity. The complementary use of OCT, OCT-A, and FA enhances diagnostic accuracy, facilitates timely therapeutic interventions, and supports individualized management. Regular ophthalmological monitoring, including advanced imaging modalities, should be considered an essential component of care in Susac syndrome.

## 1. Introduction and Clinical Significance

Susac syndrome is a rare systemic inflammatory disorder characterized by a clinical triad of encephalopathy, branch retinal artery occlusion (BRAO), and sensorineural hearing loss, primarily affecting the middle-aged population [1], with a predilection for young women and a peak incidence in the third decade of life [2]. The syndrome was first described by Susac in 1979 [3]. The exact etiology remains unclear; however, current research indicates that the pathogenesis involves autoimmune-mediated inflammatory endotheliopathy, leading to microinfarctions in the brain, retina, and inner ear [1,2].

Ocular manifestations of Susac syndrome primarily involve retinal changes, with BRAOs or arteriolar wall hyperfluorescence (AWH) observed on fluorescein angiography (FA) being the most common findings [4,5]. Other ocular findings may include cotton-wool spots, microaneurysms, and optic disc edema or atrophy [4]. The visual symptoms associated with Susac syndrome are variable and can range from mild to transient or persistent visual disturbances, manifesting as visual field defects. However, in some cases, severe and permanent vision loss may occur [4].

In this report, we present two case studies highlighting the complementary role of multimodal imaging, including optical coherence tomography (OCT) and FA, in the assessment and longitudinal evaluation of retinal vascular involvement in patients with Susac syndrome.

## 2. Cases Presentation

### 2.1. Case 1

A 43-year-old Caucasian male was referred to the Department of Ophthalmology at the University Clinical Hospital in Poznań (Poland) for an ophthalmological consultation due to a diagnosis of Susac syndrome. The patient was being treated with an oral corticosteroid (prednisolone, at a dose of 4–6 mg). His medical history was significant for mixed hyperlipidemia and impaired glucose tolerance. The patient reported that he initially presented with complaints of vertigo and nystagmus, which initially led to suspicions of Ménière’s disease and multiple sclerosis; however, both conditions were excluded. Subsequently, occlusion of the superotemporal branch retinal artery in the left eye (OS) was identified, and focal laser photocoagulation was performed in the corresponding retinal area. Sectoral laser photocoagulation was performed to reduce the risk of neovascular complications, as peripheral capillary non-perfusion was visible on FA. Based on neurological assessment, ophthalmic findings, and hearing loss identified on tonal audiometry, a diagnosis of Susac syndrome was made. On ophthalmological examination, best-corrected visual acuity (BCVA) was 0.00 logMAR for both eyes (OU) after correcting for myopia, and intraocular pressure (IOP) was within the normal range. There were no abnormalities in the anterior segment of OU. Fundus examination of the right eye (OD) revealed no pathological changes apart from a borderline c/d ratio of 0.5 of the optic disc. In the left eye (OS), the c/d ratio was also borderline, measured at 0.6. OCT RNFL measurements showed a mean global thickness of 73 µm in the OD and 84 µm in the OS. Sectoral analysis revealed superior quadrant thinning in both eyes. In OD, additional borderline values were observed inferiorly and nasally with preserved temporal thickness, whereas in OS, thinning was confined predominantly to the superior quadrant with preserved nasal, inferior, and temporal sectors.

**Figure 1:**
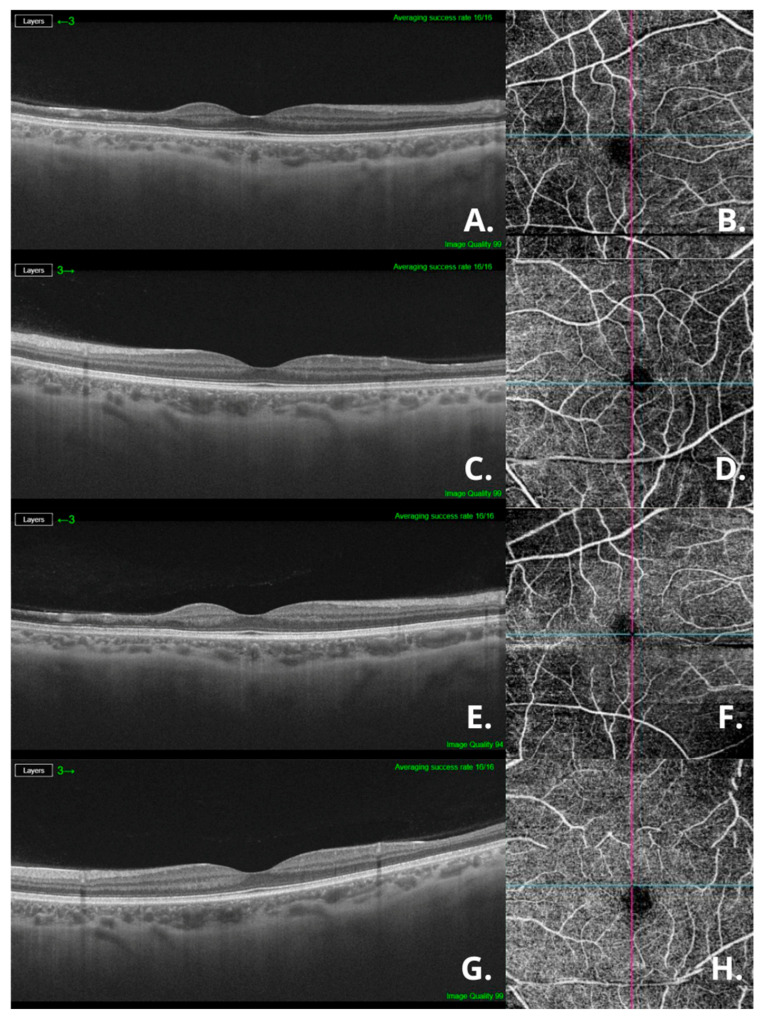
Macular optical coherence tomography and optical coherence tomography angiography scans in Case 1. (**A**). Inner retinal thinning in the temporal sector, right eye. (**B**). Decreased vessel density in the temporal and inferior areas, right eye. (**C**). Inner retinal thinning in the temporal sector, left eye. (**D**). Decreased vessel density in the temporal area, left eye. (**E**). Inner retinal thinning in the temporal sector, right eye (follow-up examination after 10 years). (**F**). Decreased vessel density in the temporal and inferior areas, right eye (follow-up examination after 10 years). (**G**). Inner retinal thinning in the temporal sector, left eye (follow-up examination after 10 years). (**H**). Decreased vessel density in the temporal area, left eye (follow-up examination after 10 years). Device: DRI OCT Triton (Topcon Corporation, Tokyo, Japan). Software: Topcon ImageNet 6 (Topcon Corporation, Tokyo, Japan). Scan area: 6 × 6 mm macular scans. OCT-A images were analyzed using the superficial and deep capillary plexus slabs. Vessel density assessment was qualitative, based on visual comparison of serial en face OCT-A images and correlation with structural OCT findings.

**Figure 2:**
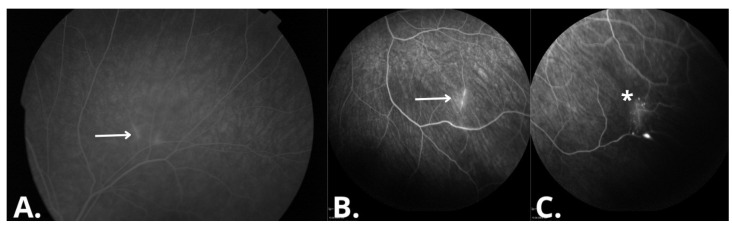
Fluorescein angiography. Characteristic symptoms of Susac syndrome. (**A**). Case 1. Arteriolar wall hyperfluorescence (AWH), left eye, peripheral superior temporal sector. Device: 50IX retinal camera (Topcon Corporation, Tokyo, Japan). (**B**). Case 2. AWH, left eye, peripheral temporal sector. (**C**). Case 2. AWH and microaneurysms, left eye, peripheral inferior temporal sector. Arrows indicate areas of AWH, and asterisk indicate microaneurysms. Device: Spectralis HRA + OCT (Heidelberg Engineering, Heidelberg, Germany).

Four years after the initial visit, the patient was admitted to the Ophthalmology Outpatient Clinic due to a new dark spot in the visual field of the OS, located in the superior temporal sector. The patient reported noticing the defect upon awakening. BCVA remained at 0.00 logMAR, and IOP was normal. Anterior segment examination revealed an initial bilateral posterior subcapsular cataract not involving the visual axis. Fundus examination showed foci of retinal swelling along the inferior temporal arcade. Macular OCT of the OD revealed an area of atrophy in the inner retinal layers in the temporal and inferior regions (Figure 1A), while the OS showed full-thickness atrophy in the temporal area (Figure 1C). OCT-A showed decreased vessel density in the corresponding localizations in the OU (Figure 1B,D). The mean RNFL thickness decreased to 69 µm in OD and 76 µm in OS. Superior quadrant thinning persisted in both eyes, with additional borderline inferior values in OS and nasal involvement in OD, indicating structural progression compared to baseline. FA revealed a focal retinal infarction in the OS, consistent with a sequela of BRAO. No neovascular changes were observed in OU. A subsequent follow-up visit three years later showed no abnormalities in the OD, while arterial wall hyperfluorescence (AWH) at the temporal-superior quadrant was shown in the OS (Figure 2A). Visual acuity remained at 0.00 logMAR for both eyes, and IOP was normal. The global RNFL thickness measured 70 µm in OD and 72 µm in OS. Compared with the 4-year examination, RNFL values remained relatively stable in OD, while a slight additional reduction was observed in OS, predominantly affecting the superior and inferior quadrants. After a further three years, the patient presented with the appearance of a crescentic spot in the superior nasal quadrant of the visual field. The patient reported that the first spot appeared five days before presentation and then disappeared, followed by a new spot in the same area three days ago, which also resolved the day before admission. BCVA remained at 0.00 logMAR, and IOP was normal. Apart from the initial posterior subcapsular cataract in OU, the anterior segment of both eyes was unchanged. No new pathological changes were visible on fundus examination in the OS; while examination revealed a new retinal microinfarction near the inferior temporal arch of the OD. There were no visible new pathological changes in the macular scan of the OU (Figure 1E,G); however, the OCT-A scan revealed further vessel density loss in the inferior area of the macula of the OD (Figure 1F), with stable vascular density status in the macula of the OS (Figure 1H). The mean RNFL thickness was 71 µm in OD and 70 µm in OS. RNFL values remained largely stable in OD, whereas OS showed borderline inferior and nasal values with preserved temporal thickness, without evidence of rapid additional global thinning. The patient was referred to the Rheumatology Clinic to intensify systemic treatment. He received three doses of intravenous methylprednisolone (500 mg) followed by oral prednisolone with gradual tapering. Mycophenolate mofetil was added to the treatment regimen, and azathioprine was discontinued. Spots in the visual field persisted; however, no new pathological changes were identified during further ophthalmological examination.

According to the diagnostic criteria of Kleffner et al. [5], Case 1 met the definition of definite Susac syndrome, as all three components of the classical triad were documented: encephalopathy, branch retinal artery occlusion, and sensorineural hearing loss.

#### Chronological Summary—Case 1

Initial presentation led to the diagnosis of Susac syndrome based on neurological findings, BRAO in the OS, and sensorineural hearing loss. Four years later, the patient developed a new visual field defect in the OS. OCT revealed inner retinal atrophy, OCT-A demonstrated reduced vessel density in the corresponding areas, and FA confirmed focal retinal infarction. At the 7-year follow-up, arteriolar wall hyperfluorescence was detected in the OS without a decline in visual acuity. Three years later, a new retinal microinfarction was identified in the OD. OCT-A revealed further vessel density loss, while BCVA remained preserved.

### 2.2. Case 2

A 31-year-old woman was admitted to the Ophthalmology Clinic at the University Clinical Hospital in Poznan (Poland) for an ophthalmological consultation due to a diagnosis of Susac syndrome during hospitalization in the Department of Neurology, where systemic immunosuppressive therapy with oral corticosteroids and azathioprine was initiated. She had undergone an ophthalmological examination two years before, and she had been prescribed spectacles for the correction of low myopia. She had a history of migraine with aura. She denied any deterioration of visual acuity. In 2014, she developed an episode of non-infectious meningitis and encephalitis with encephalopathy and cerebellar syndrome. She denied other diseases and drug intake.

**Figure 3:**
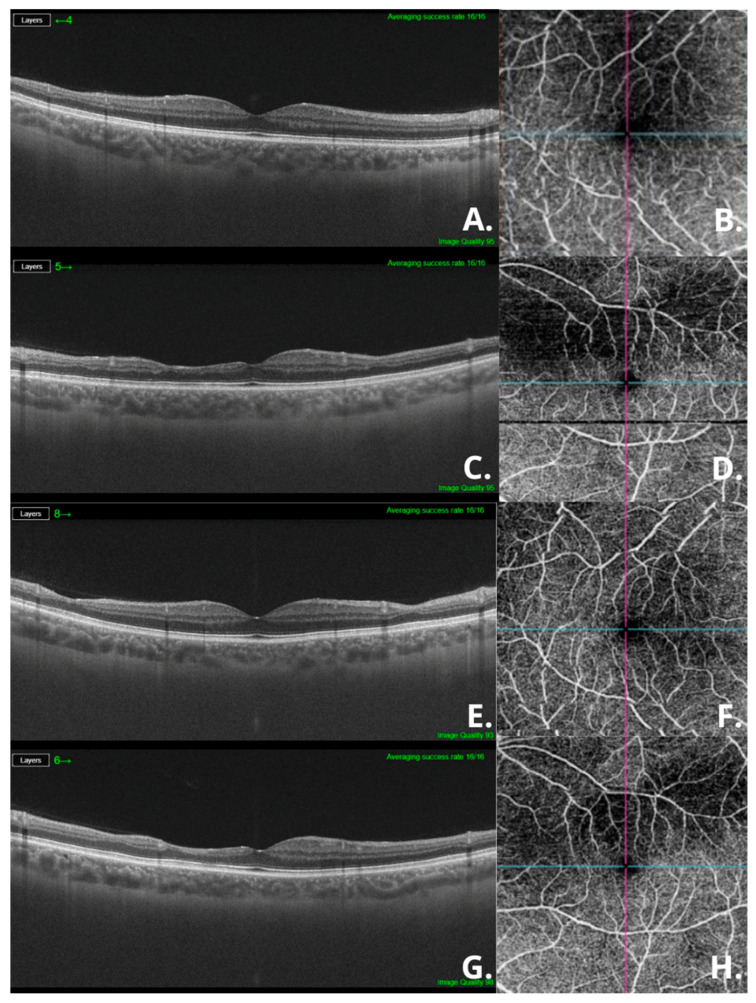
Macular optical coherence tomography and optical coherence tomography angiography scans in Case 2. (**A**). Inner retinal thinning in the superior sector, right eye. (**B**). Decreased vessel density in the temporal and inferior areas, right eye. (**C**). Inner retinal thinning in the nasal and temporal sector, left eye. (**D**). Decreased vessel density in the temporal area, left eye. (**E**). Inner retinal thinning in the macular periphery (progression), right eye (follow-up examination 2 months after presentation). (**F**). Diffuse decreased vessel density in the macular area, right eye (progression; follow-up examination 2 months after presentation). (**G**). Inner retinal thinning in the nasal and temporal sector, left eye (follow-up examination 2 months after presentation). (**H**). Decreased vessel density in the temporal area, left eye (follow-up examination 2 months after presentation).

At presentation, physical examination revealed color vision impairment in the OD and partial impairment in the OS. The BCVA was 0.00 logMAR for the OU, and the IOP remained within normal range. Slit lamp examination revealed no pathological changes in both the anterior and posterior segments of the OD and OS. OCT RNFL revealed sectoral thinning in the superior nasal and inferior quadrants of the OD, and in the superior and inferior quadrants of the OS, with global mean thickness values of 65 µm and 68 µm, respectively. Ganglion Cell Layer (GCL) examination showed generalized thinning, particularly in the nasal quadrant of the OD and borderline values at the inferior quadrant of the OS. Macular OCT of the OD revealed inner retinal thinning in the superior and temporal sectors (Figure 3A), and in the superior sector of the OS (Figure 3C). OCT-A revealed a decreased density of vessels in the corresponding areas of the OU. FA showed peripheral AWH in the OU, and microaneurysms in the temporal-inferior part of the OS (Figure 3B,D).

At the first follow-up (approximately two months after baseline), macular OCT revealed inner retinal thinning in the peripheral macular sectors with new focal thinning in the nasal sector of the OD (Figure 3E), and in the superior part of the macula of the OS (Figure 3G). OCT-A showed diffuse decreased vessel density in the macula of the OD (Figure 3F), and in the superior sector of the macula of the OS (Figure 3H). OCT RNFL revealed thinning in the upper and lower quadrants of the OU, with a mean RNFL thickness of 69 μm and 70 μm for OD and OS, respectively. Although the global mean RNFL thickness showed minimal fluctuation between visits, localized thinning in the superior and inferior quadrants was evident on sectoral analysis. These changes were interpreted as true regional atrophy rather than measurement variability. A subsequent examination four months later revealed no further disease progression. The patient denied any visual symptoms and was referred for a control visit in three months.

According to the diagnostic criteria proposed by Kleffner et al. [5], Case 2 met the definition of definite Susac syndrome, as the patient presented with the complete clinical triad—encephalopathy with ischemic lesions on MRI, branch retinal artery occlusion, and sensorineural hearing loss.

#### Chronological Summary—Case 2

At baseline, the diagnosis of Susac syndrome was established in the presence of BRAO, sensorineural hearing loss, and MRI-confirmed encephalopathy. Two months later, progression of localized inner retinal thinning and reduced vessel density on OCT-A was observed in OU. By the 4-month follow-up, no further disease progression was detected, and retinal imaging findings had stabilized under systemic immunosuppressive therapy.

## 3. Discussion

Susac syndrome is a rare autoimmune disease characterized by a triad of symptoms—encephalopathy, BRAO, and sensorineural hearing loss secondary to microinfarctions in the precapillary arterioles of the brain, retina, and the cochlea and semicircular canals of the inner ear [6]. Both presented cases fulfilled the diagnostic criteria for definite Susac syndrome according to Kleffner et al., exhibiting the characteristic triad of encephalopathy, branch retinal artery occlusion, and sensorineural hearing loss. Recognition of this triad remains essential for timely diagnosis and differentiation from other demyelinating or autoimmune vasculopathies [5].

Despite advancements, the pathophysiology of Susac syndrome remains unclear. It is believed to primarily affect the endothelial cells of precapillary arterioles in the brain, retina, and inner ear [7]. The leading hypothesis is that endothelial cell damage is secondary to the action of anti-endothelial cell antibodies (AECAs); however, in a study by Jarius et al., AECAs were present in only 25% of patients with Susac syndrome; therefore, their pathogenic role remains uncertain [7,8].

The time from the onset of the first symptoms to the full manifestation of the disease differs between individual cases. Components of the triad are present simultaneously in only 13–20% of patients at the time of presentation [1,9]. The most common manifestation of Susac syndrome is CNS and ocular involvement, observed in 60% of patients, compared to audiometry changes found in about 30% of individuals [10]. In both cases presented, the diagnosis was established following CNS and ocular involvement. The dynamics of their presentation were also gradual. Initially, the symptoms presented were less specific, and only the appearance of more characteristic symptoms, especially BRAO at a young age, led to the diagnosis of Susac syndrome being considered.

Headaches can be one of the first symptoms of Susac syndrome and are a sign of central nervous system involvement [11]. The disease is preceded by migraine-like headaches in more than 80% of cases [1], as we described in our second case, where headaches accompanied by visual disturbances were initially misdiagnosed as migraine with aura. Moreover, according to a study by Cohen et al., headaches were present in 95% of patients with Susac syndrome [12].

There is limited data on patients’ demographic characteristics due to the rarity of the disease. Retrospective studies have shown that the majority of patients were between 20 and 40 years old, although there have also been reports of Susac syndrome diagnosis in patients aged 8 to 72 [6,13]. It has been shown that age at presentation can determine the clinical course of the disease, with a tendency toward a more severe course of the disease with rapidly deteriorating vision and hearing loss in younger patients [14]. Caucasian individuals predominate among the patients, as was the case with our cases; however, cases of Susac syndrome in individuals of Asian, African, and Hispanic descent have also been reported [15]. The disease shows a clear female predominance, with women representing approximately 70–80% of reported cases [16]. The underlying mechanisms for the higher prevalence of Susac syndrome in the female gender are not fully understood; however, the influence of hormonal or immunological factors modulating the autoimmune response is strongly suspected. Some reports suggest that estrogen may have a role in the pathogenesis of the disease, which may explain the higher prevalence in females [5].

Visual impairment in patients with Susac syndrome is often due to BRAO or vasculitis, which patients frequently report as an altitudinal defect, central or paracentral scotoma, or, less commonly, flashes in the visual field [7]. In more severe cases, loss of a larger visual field area or complete vision loss may occur due to central retinal artery occlusion (CRAO) [9]. On the other hand, in rare cases, an embolism occurring at the far periphery of the retina may not cause visual symptoms to the patient [7]. Visual acuity (VA) in patients with Susac syndrome is usually unaffected. In a study by Cohen and coworkers, the median VA of patients with Susac Syndrome at the first and final visit was 20/20 in OU [12]. Similarly, in both cases presented, VA was 0.00 logMAR during follow-up. Our male patient (Case 1) reported periodic new paracentral visual field scotomas in the OD and OS, which resulted from new episodes of BRAOs. In contrast, our female patient (Case 2) reported no visual symptoms.

Typical findings during fundoscopy in patients with Susac syndrome include Gass plaques, yellowish-white lesions localized within the retinal arterial walls, which are visible during fundoscopy and can be misdiagnosed as embolic material. Gass plaques result from an autoimmune reaction occurring in the walls of retinal arterioles. Gass plaques can localize throughout the course of retinal arterioles [7]. Importantly, Gass plaques usually appear in the acute phase, while they may disappear in the stable phase of the disease [7]. This phenomenon may explain the absence of Gass plaques in our patients during the study, even in our Case 1, whose disease continued to show low to moderate activity despite the appearance of new visual complaints during follow-up.

Other characteristic retinal lesions include areas of retinal whitening, which is an early sign of ischemic episodes, neovascularization, or secondary vitreous hemorrhages [7]. In our Case 1, peripheral sectoral laser photocoagulation of the retina was performed in the OU due to ischemic changes found in the FA to prevent complications secondary to neovascularization.

Ocular imaging modalities, including OCT, OCT-A, and FA, play a crucial role in diagnosing, monitoring, and managing patients with Susac syndrome, facilitating the detection of both remission and relapses [7]. RNFL thinning is one of the hallmark lesions found on OCT in patients with Susac syndrome and is reported to be present in 68% of patients [4]. Atrophy of the inner retinal layers, including the RNFL, GCL, inner plexiform layer (IPL), and inner nuclear layer (INL), is also a distinctive finding and reflects the chronic stage of retinal ischemia [7]. Notably, due to choriocapillaris supply, the outer retina and retinal pigment epithelium (RPE) are typically unaffected [7]. If microinfarctions occur in the foveal or perifoveal region, macular OCT may show flattening of the foveal contour, which aids in differentiating Susac syndrome from multiple sclerosis (MS) and other demyelinating diseases. Despite regular Humphrey Visual Field (HVF) results, OCT can present abnormalities, demonstrating its superior diagnostic sensitivity [17].

Brandt et al. showed that patients with Susac syndrome are characterized by RNFL thinning and a reduction in the total macular volume (TMV) compared to healthy individuals and patients with relapsing-remitting multiple sclerosis (RRMS) without previous episodes of optic neuritis, but not in RRMS individuals with a history of optic neuritis, in whom TMV was also decreased [18]. Similarly, in a study by Ringelstein et al., areas of the retina previously affected by ischemic events in patients with Susac syndrome were characterized by the reduced thickness of the RNFL, GCL, IPL, INL, and outer plexiform layer (OPL) compared to healthy individuals and RRMS patients, both with and without a history of optic neuritis. In contrast, there were no differences in the thickness of the ONL and IPL layers between RRMS and healthy individuals. In the Susac syndrome group, in retinal sectors unaffected by episodes of microinfarctions, the RNFL and IPL layers were thinner compared to the healthy individuals, but only the IPL was significantly thinner in Susac syndrome patients compared to the RRMS group without a history of optic neuritis. At the same time, the RNFL and GCL were thinner in RRMS patients with a history of optic neuritis [19].

OCT-A is a non-invasive imaging technique that visualizes motion contrast signals generated by moving red blood cells, enabling reconstruction of retinal microvasculature without the need for contrast agents. Unlike fluorescein angiography, OCT-A does not visualize vascular leakage but rather depicts perfused vessels based on flow signal [20]. OCT-A detects retinal capillary loss areas at the posterior pole and, in a wide-field modality, also at the retinal periphery [20]. The advantage of OCT-A over FA is its ability to accurately image both superficial and deep retinal vascular plexuses, thereby detecting areas of vascular non-perfusion [21]. In the present report, OCT-A analysis was performed qualitatively rather than quantitatively, as numerical vessel density metrics were not available within the applied software version. The Topcon DRI OCT Triton system (ImageNet 6 software) used in this study provides qualitative assessment of vascular perfusion maps without automated quantitative vessel density metrics. Structural OCT parameters, including global and sectoral RNFL thickness, were quantitatively measured to support interpretation of microvascular alterations. Nevertheless, serial visual comparison of corresponding en face slabs allowed reliable identification of vascular rarefaction and progression. Future studies with quantitative OCT-A tools may better define microvascular changes in Susac syndrome.

FA is a valuable tool for managing patients with Susac syndrome. Current studies have revealed that FA possesses higher sensitivity in detecting Susac syndrome activity than MRI and audiometry. In a survey by Guttieres and coworkers, disease activity was detected in 41.5% of FA examinations, 10.5% of MRI scans, and 25% of audiograms [10]. Similarly, in another study, among 11 episodes of Susac syndrome activity, FA showed activity in 9 cases (81.8%), which was the highest detection rate compared to audiograms (72.7%) and MRIs (18.2%). Interestingly, visual field and fundus examinations were allowed to detect signs of disease activity in only 36.4% of patients, emphasizing the importance of FA in diagnosing and follow-up of patients with Susac syndrome [12].

FA enables the detection of characteristic lesions in Susac syndrome, including AWH, arterial leakage, microinfarctions, and microaneurysms, or BRAOs, in contrast to indocyanine angiography (IA), which is usually normal [7]. On FA, AWH is a typical feature in patients with Susac syndrome, and this sign was present in both of the cases described. This finding can occur peripherally from the occluded and unaffected vessels. Notably, the presence of AWH localized away from the BRAO is a highly characteristic finding in Susac syndrome and may strongly suggest disease activity. Interestingly, AWH can be observed on FA even after clinical symptoms have resolved, indicating ongoing subclinical disease activity [7]. Additionally, in our case, two other findings mentioned above, such as retinal microaneurysms, were also present. These abnormalities in Susac Syndrome were reported in 2022 by Zur et al., and their presence is probably related to pericyte damage, AECAs-mediated endothelium disruption, and increased vascular endothelial growth factor production due to retinal ischemia [4].

In a study by Cohen and coworkers, BRAO was present in 10 (50%) patients with Susac syndrome during follow-up, while recurrent BRAO was found in 7 cases [12]. In our Case 1, we also observed recurrent BRAO in the OD after the BRAO episode in the OS, which occurred between ischemic events in the OD. The incidence of bilateral BRAO in patients with Susac syndrome has not yet been determined. In a study by Brandt and coworkers, 1 of 9 patients developed bilateral BRAO during follow-up [18]. In the study mentioned above, FA also proved to be a more sensitive marker of recurrence than audiograms, medical history, physical examination, and MRI [12], as noted in a study by Guttieres et al. FA showed an average of 2.5 leakages in both eyes during relapse compared to 1.2 leakages during remission [10].

These findings highlight the greater sensitivity of FA over physical examination and underscore the importance of performing FA in all patients with suspected or confirmed Susac syndrome, even in the presence of normal fundoscopy findings [7].

Wide-field OCT-A may be considered an alternative imaging modality to FA in patients with Susac syndrome [10]. The primary advantage of OCT-A lies in its non-invasive nature; however, this imaging modality provides a structural and flow-based visualization of the microcirculation, and therefore, cannot detect inflammatory changes affecting the arteriolar wall, which are essential markers of Susac syndrome activity [10,21]. Nevertheless, FA reflects the current vascular status at the examination, which may not capture transient episodes of BRAO and AWH. In contrast to FA, OCT technology also enables the evaluation of retinal tissue structure, allowing for the detection of signs of previous retinal ischemic episodes, such as inner retinal thinning [19]. Therefore, all mentioned modalities should be used in a complementary manner due to their distinct properties and diagnostic capabilities.

### Clinical and Scientific Contribution of the Present Cases

The present report adds to the existing literature by providing longitudinal multimodal documentation of retinal vascular changes in Susac syndrome. In particular, our observations demonstrate progressive OCT-A–detected microvascular alterations despite preserved BCVA during follow-up, alongside FA evidence of ongoing subclinical disease activity. These findings underscore the importance of multimodal, longitudinal imaging even in clinically stable patients and highlight the complementary role of OCT, OCT-A, and FA in detecting structural and vascular changes not reflected by BCVA examination alone.

## 4. Conclusions

Ophthalmological examination is a crucial component for establishing a diagnosis and monitoring disease activity in patients with Susac syndrome. The use of currently available multimodal imaging techniques in the management of patients with Susac syndrome, such as OCT, OCT-A, and FA, is beneficial due to the different diagnostic properties and capabilities of these imaging methods. Further research is needed to integrate multimodal imaging more effectively into clinical practice, improve care strategies for patients with Susac syndrome, and develop individualized management algorithms.

## Data Availability

The data (including anonymized clinical images) supporting the findings of this study are available from the corresponding author upon reasonable request.

## Abbreviations

The following abbreviations are used in this manuscript:

AECAs	Anti-endothelial cell antibodies
AWH	Arterial wall hyperfluorescence
BRAO	Branch retinal artery occlusion
BCVA	Best-corrected visual acuity
CNS	Central nervous system
FA	Fluorescein angiography
GCL	Ganglion cell layer
HVF	Humphrey Visual Field
IOP	Intraocular pressure
INL	Inner nuclear layer
IPL	Inner plexiform layer
MS	Multiple sclerosis
MRI	Magnetic resonance imaging
OD/OS	Oculus dexter (right eye)/oculus sinister (left eye)
OU	Oculus uterque (both eyes)
OPL	Outer plexiform layer
OCT	Optical coherence tomography
OCT-A	Optical coherence tomography angiography
RNFL	Retinal nerve fiber layer
RRMS	Relapsing-remitting multiple sclerosis
TMV	Total macular volume
VEGF	Vascular endothelial growth factor
